# Assessment of Darkling Beetle Fauna after Implementation of an Environmental Restoration Program in the Southern Iberian Peninsula Affected by the Aznalcóllar Toxic Spill

**DOI:** 10.1673/031.011.5801

**Published:** 2011-05-04

**Authors:** Ana M. Cárdenas, José L. Bujalance, Juan M. Hidalgo

**Affiliations:** ^1^ Department of Zoology, University of Córdoba. Campus Universitario Rabanales. Edif. Darwin. E-14071-Córdoba, Spain; ^2^ Pl. Blas Infante, I. 14850 BAENA, Córdoba, Spain; ^3^ Department of Zoology, University of Córdoba. Campus Universitario Rabanales. Edif. Darwin. E-14071-Córdoba, Spain

**Keywords:** Coleoptera, Edaphic insects, Environmental recovery, Guadiamar, Tenebrionidae, mine accident

## Abstract

This study is part of the Follow up Restoration Program of animal communities that colonize the Guadiamar River Basin. In 1998, the area was affected by a release of toxic sludge after the retention walls of the Aznalcóllar Mines (southern Iberian Peninsula) broke. The main objective of this study was to assess the current state of the population of Tenebrionidae, one of the most representative groups of edaphic Coleoptera inhabiting the Guadiamar River Basin. This paper analyses the progress made by the darkling beetle community six years after the disaster occurred and the Restoration Program was implemented. The study is based on faunistic data from systematic sampling carried out for six years to monitor plots distributed across the damaged area. To make an overall assessment of the tenebrionid fauna in relation to adjacent areas qualitative and quantitative ecological indices were applied, and temporal follow up and biogeographical comparisons were also made. The results indicate that, on the whole, tenebrionid fauna was somewhat affected by the Aznalcóllar Mine spill, and that a greater loss of fauna was detected closer to the accident site. The analysis of the temporal population dynamic suggests that the most affected zones are undergoing a process of re-colonization. However, this process varies widely by species and has not yet reached the expected levels of a non-affected river basin in the southern Iberian Peninsula.

## Introduction

In the spring of 1998, the retention walls in a tailing pond of the Aznalcóllar mine ruptured, releasing an estimated 6 Hm^3^ of sludge containing pyrite and acidic waters into the Guadiamar River basin. The toxic waste inundated the floodplain, covering an area of 4600 ha over a 62 km tract with a depth of up to 1.5 m of sludge. The toxic sludge was diverted by a containing dyke just before reaching the boundaries of the Doñana National Park, declared a Biosphere Reserve by UNESCO in 1980 (Anguas et al. 2002).

The authorities immediately undertook a series of emergency measures to clean up and remove the sludge and set a plan in motion for the integral management of the river basin known as PICOVER, the Green Corridor and Guadiamar Restoration Project (Proyecto de Restauración del Corredor Verde y del Guadiamar). This plan included the remediation of degraded fluvial and terrestrial ecosystems together with the rehabilitation of the affected zone as an ecological corridor between two singular ecosystems: the Sierra Morena Mountains and the Doñana National Park (Cárdenas and Hidalgo 2006, 2007). The main sludge removal operations and courses of action undertaken to assist the restoration of the Guadiamar basin are described in PICOVER (2003). These initiatives were based on the environmental connectivity of protected areas in the Mediterranean basin (Castro 2003; García-Montes 2003).

In 2004, six years after the accident, a new program named SECOVER, Eco-Regional Monitoring of the Guadiamar Green Corridor (Seguimiento Ecorregional del Corredor Verde del Guadiamar) was put into practice to assess whether the management of the affected area had indeed led to a environmental regeneration process and to design a long-term follow-up plan for the Green Corridor (Montes and Carrascal 2008).

The present study is framed in the abovementioned SECOVER plan and examines changes to the darkling beetle (Coleoptera, Tenebrionidae) fauna after the implementation of the PICOVER project. Research is focused on these insects because they constitute a significant group within the soil macro-fauna and are highly susceptible to the effects of environmental disturbances because they are wing-less and have limited dispersion power. The close relationship between these coleopterans and soil conditions is enhanced by the fact that the larvae of many of the species are hypogeal thus making them highly vulnerable to soil pollution. As a consequence, any alteration in the edaphic system can induce changes in the composition and structure of the darkling beetle communities (Lavelle et al. 1997) that often lead to a loss of biodiversity.

In addition, Tenebrionids are well represented, highly abundant and diverse in Mediterranean ecosystems, where they play an important role in two different respects: as detritivorous insects, taking part in the recycling and transformation process of soil (Crawford 1979, 1991; Crawford and Taylor 1984) and as prey, forming a noteworthy part of the diet of many vertebrate predators (Cárdenas and Hidalgo 2006; Bujalance 2008). Moreover, darkling beetles are considered to be good bioindicators (de los Santos 1983; Favila and Halffter 1997; de los Santos et al 2002) that makes them a useful tool to be included in environmental quality programs. Consequently, many measures of soil restoration could be assessed by following the spatial and temporal variation in tenebrionid communities.

This paper analyses the progress made by the darkling beetle community six years after the disaster occurred and the SECOVER program was implemented in order to assess the recovery level reached in area. The study is based on faunistic data taken during six years monitoring plots distributed across the damaged area. To make an overall assessment of the Tenebrionid fauna, qualitative and quantitative ecological indices were applied including measures of richness, diversity and eveness, for 6 years. Biogeographical comparisons were also made. To evaluate the level of recovery achieved in the affected zone in relation to adjacent areas, the results were compared with data from another external, ecologically similar area, the Bembézar River basin (Cárdenas and Bujalance 1985) that was unaffected by the toxic waste.

## Materials and Methods

### Study area

A comprehensive description of the study area can be found in Cárdenas and Hidalgo (2006). The Guadiamar River is located in the southern Iberian Peninsula and is one of the chief tributaries of the Guadalquivir River. The basin is approximately 65 km long and despite this short course it constitutes a true functional system between two interconnected natural areas (Montes 1999), the Doñana National Park and the Sierra Morena Mountains, both of which are of great ecological value. Detailed information concerning the abiotic features of the Doñana National Park and Sierra Morena Mountains can be found in García-Canseco et al. (2002) and Cárdenas and Bach (1988a, b, 1989a) respectively.

Particularly in the Guadiamar area, the geological horizon of the upper reaches of the basin contains sulphide-rich mineral deposits, which support the mining at Aznalcóllar. The predominant vegetation in the area consists of open oak meadowlands alternating with pine forests and eucalyptus groves, as well as shrubs and scrubland. As a consequence of intensive human intervention, few of these formations remain in the middle reaches of the basin, having been replaced by cereal, cotton, oleaginous and fruit crops, while rice fields and unmanaged marshlands predominate in the lower reaches.

From a climatic point of view, the Guadiamar basin belongs to the sub-humid, Mediterranean type, with an annual rainfall of approximately 600 mm (Marvizón and Fernández-Caro 1981).

Abiotic data related to the Bembézar River, which is considered a control area, can be found in Cárdenas and Bach (1989b).

### Sample design

PICOVER established 22 monitoring plots that were common reference areas for the multidisciplinary group involved in this research. These monitoring plots were considered to be representative of the different morphological and eco-dynamic conditions of the Guadiamar river system, including both water and soil sampling stations. Comprehensive information about the area and the study sites can be found in Montes et al. (2003).

**Figure 1.**
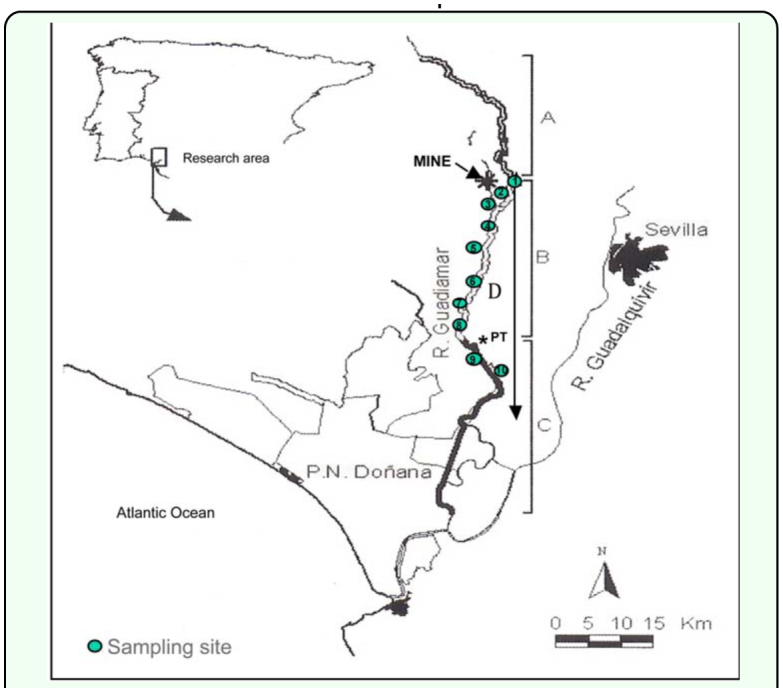
Map of the research area. A Upper reaches, B: medium reaches, C: lower reaches and D: Damaged area (from Cárdenas and Hidalgo 2006). High quality figures are available online.

Ten of these monitoring plots were selected to perform the studies on soil fauna. Figure 1 shows the layout of these sites and more detailed data concerning the location can be found in Table 1. As far as possible, site locations were chosen at regular intervals along a 60 km stretch of the disturbed riverbed, starting at the spot closest to the spillage (sampling site 1, see map).In addition, a sampling site named toxic plot (PT), from which toxic sludge was not removed during the clean-up operations, was also sampled in order to determine the persisting effect of the toxic sludge over the darkling beetle fauna.

Between March 2000 and September 2001 (first research project, PICOVER), each sampling station was visited twice a month to set and remove pitfall traps. Eight pitfall traps were set at each sampling site. The traps were active for 10–14 days depending on the weather conditions. The pitfall method is widely used in ecology for descriptive and functional studies of soil arthropod populations (Grüm 1980; de los Santos et al. 1982, 2002; Fenologio et al. 2007) and they are particularly appropriate for ground-living beetles when the aim is to compare diversity indices estimation (Henderson 2003). The traps consisted of cylindrical containers with a capacity of 1000 cc and were buried up to the top end and partially covered to prevent flooding. The traps were baited with approximately 250 cc of commercial acetic acid.

Later, between March 2004 and September 2005 (second phase, SECOVER), similar sampling procedures were used at the same sampling sites in order to make feasible comparisons and detect changes in the tenebrionid fauna that could indicate whether, in fact, the restoration actions did achieve regeneration of the area. Description of sampling stations from Bembézar river basin is described in Cárdenas and Bach (1989b).

**Table 1.**
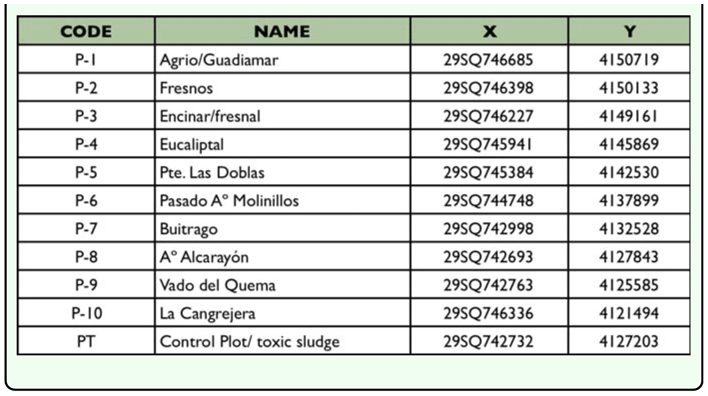
UTM Co-ordinates for the sampling sites

### Ecological methods

In order to characterise the dark beetle community, the same indices were applied in both research periods (PICOVER and SECOVER). These indices are those most commonly used in the study of insect populations (Ludwig and Reynolds 1988; Southwood 1991): richness, diversity and evenness *vs.* dominance.

To measure richness, the number of species was recorded and the following richness indices were used: Margalef's richness index R1


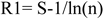


and Menhinick's richness index R2 where


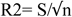


where n= individuals observed and S=species number.

To measure diversity several indices were used following the criteria defined by Ludwig and Reynolds (1988):

Shannon index


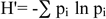


where pi= proportional abundance of each species

Hill's diversity numbers N1


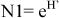


and N2


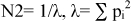


where pi= proportional abundance of each species

Evenness/dominance was measured using Hill's ratio as suggested by Lyons (1981):


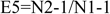


These parameters are suitable to be used for comparative purposes, especially if sampling methods, area size and sample size are similar (Southwood 1991), as in this case.

To evaluate the level of recovery achieved in the affected zone the SECOVER results were compared with data from an external, unaffected but ecologically similar area, the Bembézar River basin, another tributary of the Guadalquivir River. The Guadiamar and the Bembézar are 150 km apart at the mouth and share similar environmental features except for the mine spill. In addition to their similarities, Bembézar was selected as a control area because data concerning the area's darkling beetle populations are available (Cárdenas and Bujalance 1985). For this purpose, the rarefaction curves (Hurlbert 1971) were calculated as the most adequate method to allow comparisons when sample sizes vary due to different sampling methods having been applied. Details about this statistical procedure can be found in Ludwig and Reynolds (1988).

### Statistical analysis

In order to assess the effect of the distance from the spill on the tenebrionid beetles, the Overall area affected was divided into five sectors, approximately 10 km in length, each containing two sampling sites. Affinity between sectors was established using hierarchical cluster analysis. The Jaccard similarity coefficient was applied to the qualitative matrix data to obtain the corresponding dendrogram. This procedure considers the presence/absence of species and measures the extent to which different habitats have common species (Southwood 1991). It was applied to both the PICOVER and SECOVER sampling periods, and to both datasets when considered jointly, which should indicate changes over time/space. Calculations were performed using the PS Statistical software (SPSS Inc. 2002). To determine relationships between diversity indices and distances to the toxic spill, the Pearson product-moment correlation (Ludwig and Reynolds 1988) was applied.

**Table 2.**
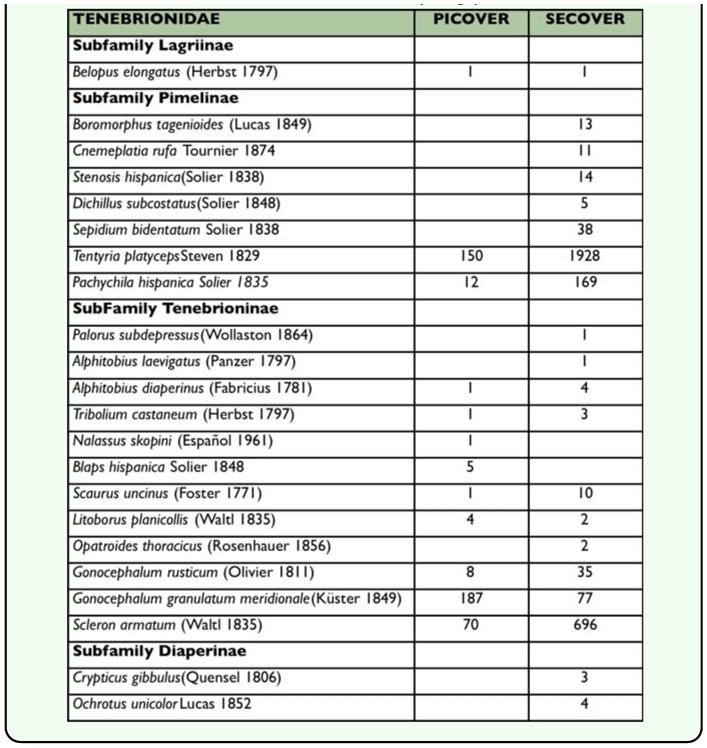
List of Tenebrionid species and their respective caught data from PICOVER and SECOVER sampling periods

### Taxonomical and Biogeographical criteria

With respect to the taxonomical treatment of the species Gebien (1937, 1938–1942a, 1942b–1948) was followed and for higher taxa (Subfamilies, Tribes and Sub-tribes) Bouchard et al. (2005, 2007), Doyen (1972) and Doyen and Lawrence (1979) were used. The Holdhaus' criteria (1929) were followed when assigning the tenebrionid species to different biogeographical categories. Comparisons from a biogeographical point of view provide evidence of significant changes in the composition of the fauna in the disturbed and/or restored areas.

**Table 3.**
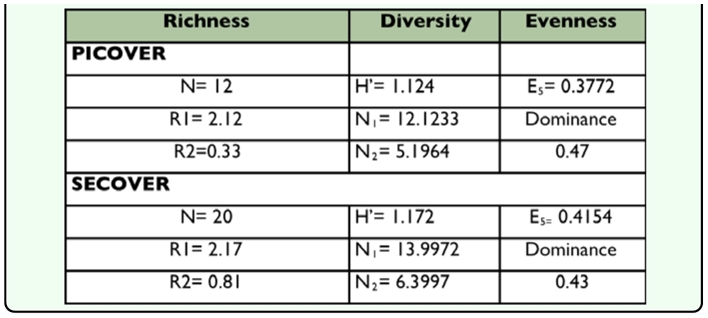
Dominance, Richness, Diversity and Evenness indices for each sampling period: N (species number), R1 (Margalef), R2 (Menhinick), H' (Shannon), N1 and N2 (Hill's Diversity) and E5 (Hill's Evenness)

## Results

### Evolution of the Darkling Beetle community

Table 2 shows the species and their respective abundance corresponding to each research period, PICOVER and SECOVER. According to the results from PICOVER (Cárdenas and Hidalgo 2006), there were signs of a noticeable impoverishment of the fauna in the affected area after the disaster that was colonized at the time of this first study by only 12 species with most of them having only a scarce or accidental presence (a very low number of specimens were recorded). Only a few generalist species, *Gonocephalum rusticum, Scleron armatum* and *Tentyria platyceps,* were abundant in the studied zone after the accident occurred and were found to be present even in the plot where the toxic sludge was not removed. This result was also supported by the comparative biogeographical analysis made of the fauna from the Guadiamar affected area and those from the two unaffected adjacent natural areas: Sierra Morena (the northernmost boundary) and Doñana National Park (in the far south) (Cárdenas and Hidalgo 2006).

The four years from the mine accident to the end of the latest restoration program in the area, were long enough to make a comparison between the sets of data corresponding to each sampling period (PICOVER and SECOVER) to assess changes in the dark beetle fauna.

In the following phase (SECOVER) 20 species of Tenebrionidae were recorded (Table 2). It may be seen that there is a persistence of certain predominant species, *T. platyceps* and *S. armatum,* that are highly competitive in arid environments. But, together with these two, other species were also found: *G. granulatum,* a more or less ubiquitous species in the overall area, and *Pachychila hispanica,* which is also highly abundant but more dispersed in the lower reaches of the damaged area. These complete the list of better represented species in this protected space. In contrast to these, half of the listed species, whose catch data are lower than five specimens, must be considered sporadic. These results indicate that the darkling beetle community still shows a marked structural simplicity and that the diversification process is still incipient. Finally, the absence of *Blaps hispanica* and *Nalassus skopini* in the second sampling period (SECOVER) should be highlighted.

**Figure 2.**
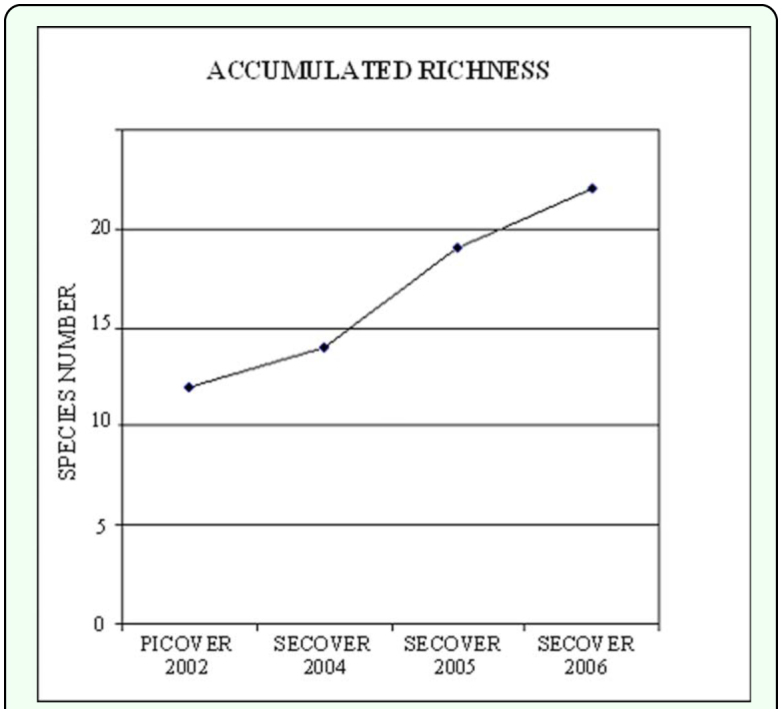
Accumulated richness in the research area. High quality figures are available online.

As shown in Table 3, despite the increase in the accumulated specific richness (plotted in Figure 2), the different indices that characterize the community (diversity, dominance, evenness) were quite similar when those of PICOVER and SECOVER were compared. It can be observed, however, that as time has passed there has been a slight increase in the values of diversity and evenness and, as expected, the effect of the dominant species has decreased.

**Figure 3.**
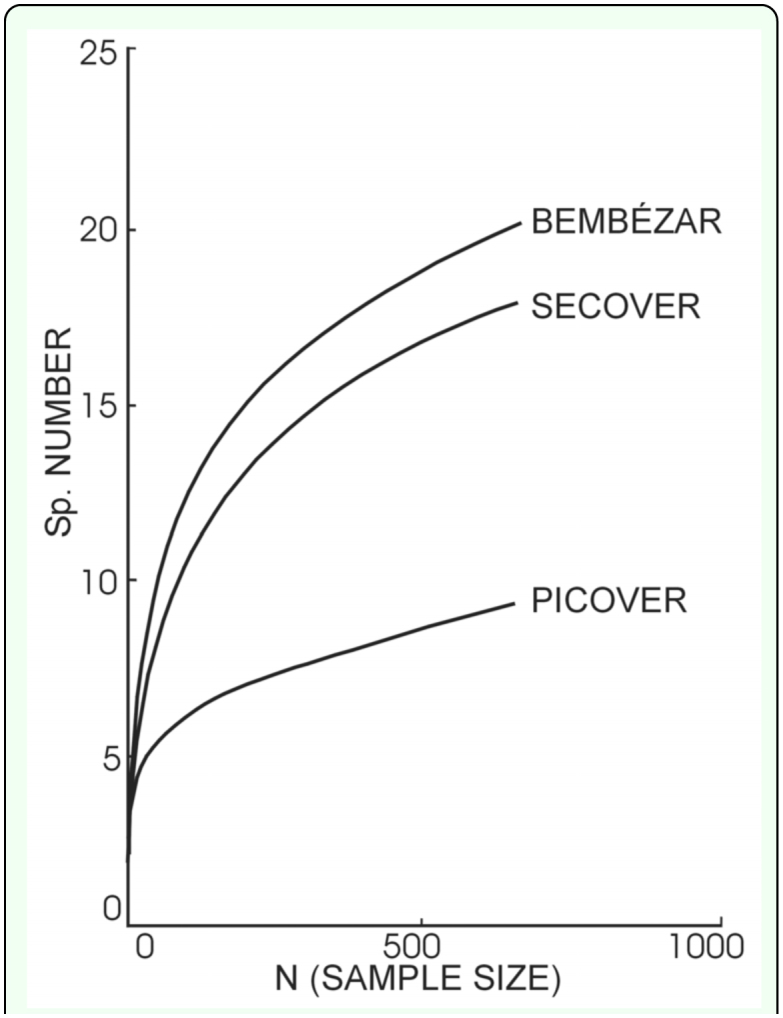
Rarefaction curves obtained for the three considered communities. High quality figures are available online.

To confirm this favourable trend, the PICOVER and SECOVER communities were compared with those from the external, unaffected area, Bembézar basin. Data corresponding to the Bembézar Basin have been taken from literature (Cárdenas and Bujalance 1985). The rarefaction curves shown in Figure 3 provides a general overview: the curve of SECOVER lies in an intermediate position between the situation detected in the PICOVER (maximum alteration) and a normal situation (the unaffected basin of Bembézar).

### Characterization by sector of the Guadiamar Basin based on Tenebrionidae fauna

By analyzing in detail each of the sectors into which the area was divided in order to assess the effect of the distance from the spill (Table 4), the results indicated that the areas farthest from the toxic spill and closest to Doñana National Park show the highest level of richness and that re-colonization in the last year has increased particularly in the more disadvantaged sections downstream. This has lead to a more uniform colonization of the area.

**Table 5.**
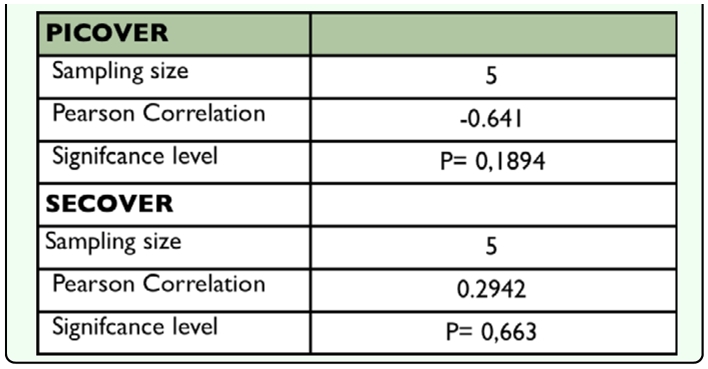
Pearson product-moment correlation between diversity indices and distances to the toxic spill

**Table 4.**
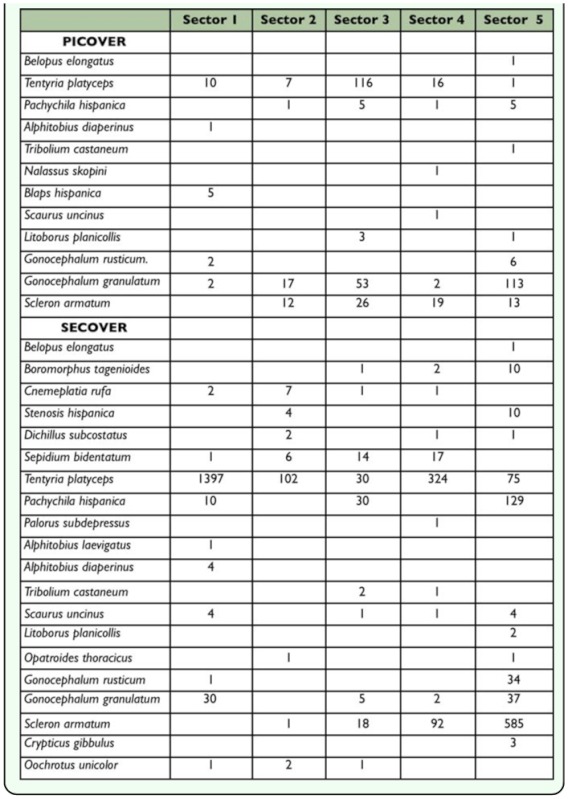
Characterization by sector of the PICOVER and the SECOVER based on Tenebrionidae fauna (specimens number)

The results also confirmed the successful expansion from Sector 5 towards the head of the basin (Sector 1). The intermediate sectors, for some time after the accident exclusively occupied by *Scleron armatum,* have already been colonized by at least ten other identified species.

The correlation coefficients calculated to assess the effect of the pollution gradient (distance from the toxic spill) on the diversity were not significant for either the PICOVER or the SECOVER datasets (Table 5).

**Figure 4.**
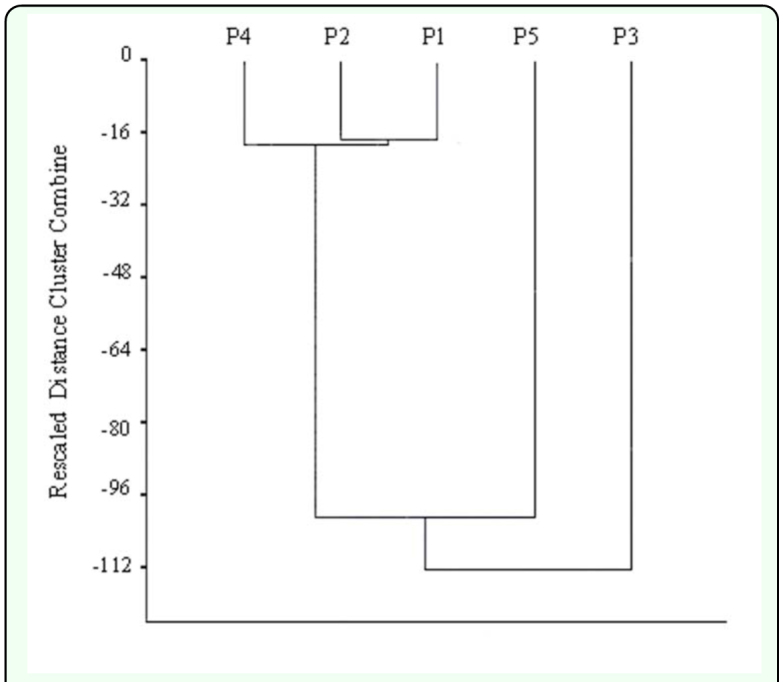
Dendrogram of species clustering of the sectors from PICOVER. High quality figures are available online.

**Figure 5.**
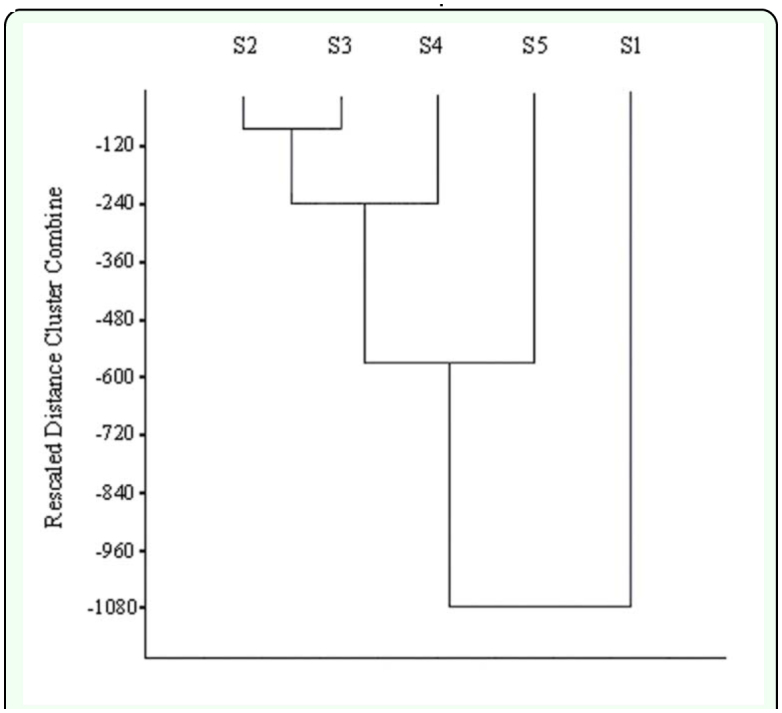
Dendrogram of species clustering of the sectors from SECOVER. High quality figures are available online.

To determine whether the effect of time has homogenised the area or, on the contrary, differences still prevail due to the distance to the toxic spill, affinity Dendrograms were constructed (Figures 4 and 5). The graphs did not show a clear trend of association for the PICOVER and SECOVER sectors when they are considered independently.

**Table 6.**
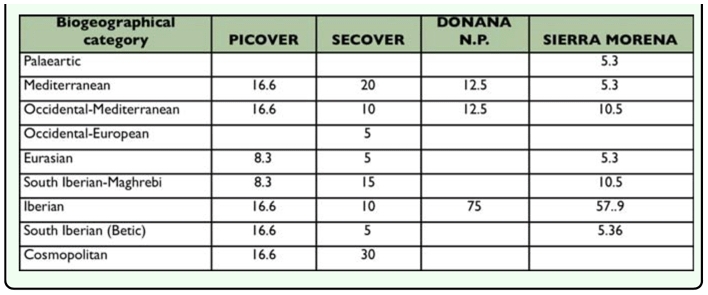
Percentage of each biogeographical element of Tenebrionidae in the affected area (PICOVER and SECOVER) and in the adjacent areas (Doñana National Park and Sierra Morena)

**Figure 6.**
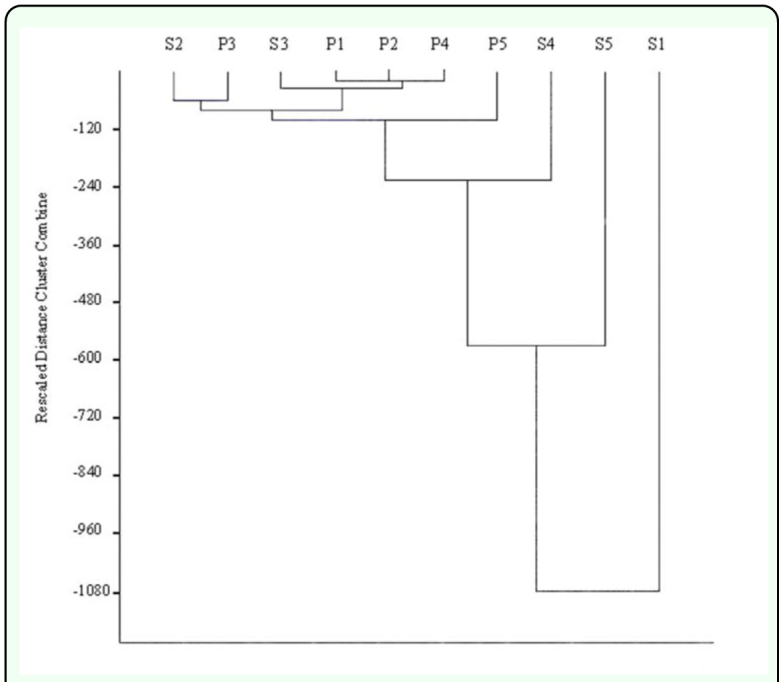
Dendrogram of species clustering of the sectors from PICOVER and SECOVER (data are jointly considered). High quality figures are available online.

The most noticeable result is the clear segregation of the sector closest to the mining spill (Sector 1) in the SECOVER phase, but this is not observed in the initial period of the PICOVER study. If the sectors in SECOVER and PICOVER are jointly considered (Figure 6), the dendrogram reveals that sectors 1 and 5 in the SECOVER phase are clearly isolated from the rest because they show the most extreme and opposite trends in the recovery process. The remaining sectors (2, 3 and 4) from the different phases of the study constitute a heterogeneous affinity group, with no clear trends of association.

### Biogeographical analysis

It is generally assumed that the ecological status of fauna in a territory can be investigated on a mesoscale by the biogeographical analysis of its elements (Vargas 1993; Davis and Scholtz 2001) and that the term “biogeographical element” refers to the taxonomic units that are characteristic of this territory. Based on these assertions, a comparative biogeographical analysis of the different research areas was also performed.

The percentage corresponding to different biogeographical categories for the darkling beetle from each research period (PICOVER and SECOVER) and data from the adjacent natural unaffected areas, Doñana National Park (Cárdenas and Hidalgo 2006), and the Sierra Morena Mountains (Cárdenas and Bujalance 1985) are shown in Table 6.

**Table 7.**
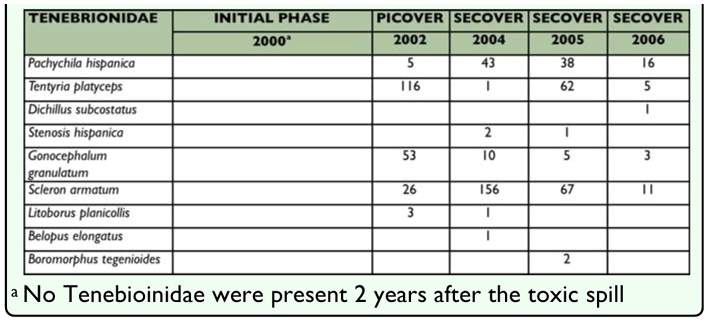
Course of the Tenebrionidae fauna in the plot PT where toxic sludge was not removed after the spill

From a chorological point of view, noticeable differences are observed between the fauna of affected and unaffected zones: while in Doñana park and Sierra Morena mountains endemisms predominate, with a fairly restricted distribution (Iberian or South Iberian), in the area under study the components with a wide distribution (cosmopolitan or Mediterranean) have a higher significance. It is also remarkable that the proportion of cosmopolitan species was double in SECOVER compared to PICOVER, to the detriment of either the betic or iberian species.

### Evolution of the Tenebrionidae Fauna in the toxic Plot

To determine the persistent effect of the toxic sludge, a detailed study on the temporal evolution of the Tenebrionidae fauna throughout all the phases of the study was also made. Data related to this aspect are shown in Table 7. In the initial phase of the study two years after the spill, the negative impact of the spill on the fauna of this plot was so severe that no darkling beetles were recorded. Later, the species number and the abundance values gradually rose until the end of 2004. From then, a gradual decrease, more evident in terms of abundance than in diversity was observed.

## Discussion

The above results provide enough information to assess the evolution of Tenebrionidae fauna after the toxic spill occurred and the restoration plan was implemented. For this research we assumed that faunistic data (qualitative and quantitative) of Tenebrionidae, that are considered to be a bioindicator group (Fávila and Halffter 1997; Cartagena and Galante 2001a, b; Cartagena 2002) within a defined habitat, enable the structure and dynamic of communities to be studied, and the impact of drastic disturbances on them to be evaluated (Southwood 1991). In practical studies, to assess biodiversity and to propose strategies towards conservation, supplementary information such as the specific richness or the endemic condition of the species must also be considered (Jaarveld et al. 1998). Therefore, the conclusions of this study are based on the comparative analysis of the indices that characterize the darkling beetle communities and their respective biogeographical composition.

An overview of the results shows that the tenebrionid populations studied around the Guadiamar River do not differ significantly from those of well-preserved natural areas like the Doñana National Park and Sierra Morena Mountains. This result was also supported by observing the respective rarefaction curves, which suggested that the accident initially had a low impact on the persistent darkling beetle populations. Similar results have also been obtained for other components of the epigean fauna (Cárdenas and Hidalgo 2006, 2007).

However, a detailed analysis of the stations closest to the river and, hence, more affected by the spill, revealed an environmental heterogeneity manifested in poorness and structural simplicity of the fauna, a process which becomes increasingly more evident closer to the site of the toxic spill. A similar gradient has also been found by Solà et al. (2003) for the fresh-water macro-invertebrates, for the amphibian communities by Tejedo and Reques (2003) from the Guadiamar River and for other coleopteran edaphic communities (Cárdenas and Hidalgo *op. cit.*).

Certain generalist species including *Gonocephalum rusticum, Scleron armatum* and *Tentyria platyceps* are not only present in the sampling plots but they are also abundant in places where the toxic sludge remains. These are species with a high environmental plasticity, capable of colonizing environments that other species are unable to tolerate. Because they are wingless, and have low dispersal ability they may have survived by taking refuge during the clean-up operations in the few areas where soil had not been removed. These ubiquitous species would then be the first agents for the re-colonization of the rehabilitated area. In the affinity analysis, the darkling beetle communities from Guadiamar and Doñana were closely linked to the studied sites because of their high proportion of common species belonging to the genera *Tentyria, Gonocephalum* and *Pimelia,* which share a thermo-xerophilous condition and preference for sandy substrates (Thomas 1983; Dajoz 2002).

The disappearance of some species in SECOVER with respect to PICOVER, such as *Blaps hispanica,* could be explained by the peculiar biology of the species belonging to this genus (Crovetti et al. 1979): 1) The long life cycle of both the larval (hypogean) and imaginai stages, that may last more than ten years in some cases (Viñolas and Cartagena 2005), and 2) Their phenology characterized by maximal activity during the spring and summer (Cartagena and Galante 2003) which coincided with the time when the mining accident occurred (April 25). In addition, being wingless, large, having low mobility and highly defined ecological requirements (particularly the vegetation) may explain their absence. The five adults of *Blaps hispanica* recorded during the PICOVER sampling could be explained as part of a residual population, heavily depleted after the ecological disaster. The absence of *Nalassus skopini* could be understood because it is a sporadic species, and so, its presence at the beginning of the study could have been merely casual.

On the other hand, biogeographical analysis reveals that the current composition of the darkling beetles community in the Guadiamar had undergone significant changes in comparison to external areas (Sierra Morena Mountains and Doñana Natural Park), although the Mediterranean component continues to be important. Furthermore, in SECOVER there is a proliferation of species with a worldwide distribution and a scarcity of elements with a more restricted distribution (mostly Iberian endemism). This could be interpreted as the accident mostly having affected the autochthonous fauna, which is always more sensitive to alterations in habitat. During the early stages of re-colonization, new opportunistic or introduced species arrived to the area, which explains the increase of the cosmopolitan element in the second period of the study.

With respect to the evolution of tenebrionids in the toxic plot, the high sensitivity of this group of insects to soil disturbance and their limited dispersal ability justifies their absence in the initial phase of the study. During the cleaning up operation (sludge removal) and the later reforestation phase, this plot offered greater stability and higher environmental humidity becoming “an area of temporary refuge”. Possibly this was the reason for an increase in representation of the species up to 2004, when the highest values of abundance were recorded. From then on, a gradual decline occurred that was more evident in terms of abundance than in diversity. Clearly, with time the surrounding areas have stabilized and, in parallel the negative effect of metal contamination has been accumulating, the toxic plot has become impoverished, retaining only reduced populations of some generalist species and other accidental species.

In short, analysing the evolution of the Tenbrionidae fauna, it can be stated that the Guadiamar basin is experiencing a slow process of regeneration. Perhaps, this process is being manifested slowly due to the intimate relationship between these beetle species and the soil. This is compounded by the hypogeal nature of the preimaginal phases in many species, which makes them very sensitive to soil contamination. Furthermore, their limited dispersal ability, compared to other groups, has also slowed the re-colonization of the area; this should however become consolidated in the longer term.

## Abbreviations

PICOVER,: Green Corridor and Guadiamar Restoration Project;
SECOVER,: Eco-Regional Monitoring of the Guadiamar Green Corridor
